# Notes of the Week

**Published:** 1921-05-14

**Authors:** 


### NOTES OF THE WEEK.

### THE NEW MINISTER AND THE BRITISH MEDICAL ASSOCIATION.

A report of a deputation of the British Medical Association to the Ministry of Health will be found on page 112. This was in connection with the move in favour of utilising some of the Poor-Law accommodation for the treatment of the ordinary public who are able to pay towards the cost of their maintenance. The new Minister, Sir Alfred Mond, being a layman, was duly instructed by Sir Clifford Allbutt and his colleagues with regard to the representative character of the British Medical Association. The feeling of the Association, as voiced by Mr. Bishop Harman, was that the general practitioner in respect of cases admitted to the infirmaries should be allowed to follow his patient within the walls and treat him there. This, as Sir Alfred Mond pointed out, might not be too easy a matter, and would depend very largely upon the good temper of the practitioner and the medical superintendent, who would constantly be in an atmosphere likely to bring about a dispute. The Minister also saw some difficulty in leaving the question of financial suitability to the doctor, and generally his remarks showed that he appreciated the salient points of the problem.

### THE B.M.A. MEETING.

We look forward to the annual meeting of the B.M.A. to be held at Newcastle during the week following July 15, under the presidency of Dr. David Drummond of Durham University. Daily sessions will be held in connection with general medicine, general surgery, pathology, and bacteriology, preventive medicine and diseases in children. On two days in the week there will be sessions in obstetrics and gynæcology, neurology, ophthalmology, laryngology, and pharmacology, and one day apiece will be devoted to dermatology, radiology, and medical sociology. The last will be presided over by Sir Jenner Verrall, one of the Direct Representatives of the medical profession on the General Medical Council.

### APPROVED SOCIETIES AND HOSPITALS.

One of the largest of the Approved Societies, namely, the National Deposit Friendly Society, is the first to acknowledge the claims of voluntary hospitals, and at its conference held at Southampton early this week agreed to devote a third of the surplus of £381,000 for the purpose of part payment of the cost of hospital treatment and nursing service to its members. The question of how this large sum will be allocated has not as yet arisen, and it is one of the principal points of the conference now taking place between the British Hospitals Association and the Approved Societies; it goes without saying, however, that whatever payment is made on behalf of a Society's members, it must be applied to their use in all parts of the country on the same plan. As it is now admitted that what suits a provincial hospital does not always suit a Metropolitan hospital, it will not be too easy a matter to arrive at a decision as to how a system of payments for insured patients can be equally applied throughout the country.

### DRASTIC REDUCTIONS AT THE LONDON HOSPITAL.

Just as we go to press the following regrettable news comes to hand. Owing to the serious financial position of the London Hospital, Lord Knutsford intimates that the Committee has decided to close two hundred beds and to cut down the out-patients by half. The patients now occupying the beds will, of course, remain until they are well enough to leave, but no more will be admitted to them. The seriousness of this position to the poor of East London is increased by the fact that there is to-day an accepted list of 761 poor people waiting for a vacant bed in the London Hospital. At present the number of out-patients dealt with annually by the hospital is about 70,000, and the reduction in this department will therefore cut off 35,000.

### A SUPER-NURSING HOME.

We are informed that a syndicate has been formed, which has the support of several well-known medical men, to start a nursing home on the best hotel lines with a capital of £250,000. Mr. Nicholas Fredericks, the retiring general manager of the Métropole and Victoria Hotels, who has been associated with the Gordon Hotels for twenty years, is the head of the enterprise, but, as an entirely new building has to be constructed, the home is not expected to open its doors this year. It will accommodate some 120 patients, to whom a charge of from fifteen guineas a week upwards is to be made. In addition to the most complete equipment of a modern hospital, the distinctive feature of this hotel-nursing-home is to be the cooking and service of food achieved by means of a combination of food and hotel experts working with the medical and nursing professions. Mr. Fredericks is an enthusiast on his subject, to which he has devoted considerable study, and his aim is that surroundings and food shall have their full value in the treatment of the patient. We await with interest further details of this project. We understand that a proportion of shares in the company will be offered for public subscription.

### COAL PERMITS FOR HOSPITALS.

The Northumberland Mine Workers' Federation Board have decided, under certain elaborate safeguards, to allow coal to be taken from the pit-heads to hospitals. In Durham, however, hospitals are not treated so kindly. The Secretary of the North Ormesby Hospital, being short of coal, sent two lorries to a colliery at East Durham; these were loaded, but about a hundred miners appeared, and, although documents were produced to prove the consignment was for the local hospital, the coal was unloaded and the lorries departed empty. Durham miners have not noticed that strikers get on so much better with the strike when they gain the sympathy of the public.

### AN UNWANTED LABORATORY.

It is hard to allow, without protest, some of the statements made in opposition to Mr. H. S. Wellcome's proposal to erect a further research laboratory at Beckenham. The profession will fully support Dr. O'Brien, Director of the Wellcome Research Laboratories in Brockwell Park, in his opinion and stated experience that disease infection of people living in the neighbourhood is not likely to occur from such a laboratory. The valuable national work carried out under his able direction of the original institution is widely known. One cannot help feeling that to criticise the whole project as a commercial undertaking is hardly just, in view of the valuable contributions to science that a variety of Burroughs and Wellcome's research establishments have already provided both at home and abroad.

### TONSILS AND ADENOIDS.

A useful purpose will be served if the protest by the Society for Prevention of Cruelty to Children against certain points in connection with the operation for removal of tonsils and adenoids results in a careful professional inquiry into the pros and cons of the subject. But, at the same time, it is not right that the idea should get abroad that this surgical work is either carelessly or unnecessarily performed at our hospitals. In our own opinion it is all nonsense to maintain that nasal douching, open-air and physical exercises will cure the extreme cases of adenoids all too common in hospital practice. That these means adopted earlier might have prevented the growth of the adenoids is quite another matter, and one over which the surgeons criticised have not the least control.

### THE PRINCE'S PORTRAIT.

The President of King Edward VII. Hospital, Windsor, is the Prince of Wales, who has now presented his portrait to the institution, where it will be hung in the board-room. We have often remarked that the voluntary system depends upon a living tradition, which is best conserved by portraits and similar artistic memorials of the men of the past who worked within its walls. Board-rooms should be, among other things, small portrait galleries.

### HOSPITALS FOR THE MIDDLE CLASS.

Sir Ernest Cassel's gift of £225,000 to found a hospital for middle-class patients with nervous diseases who cannot afford the fees of nursing homes has attracted attention mainly because of the class which it seeks to benefit and less in regard to the medical benefits and knowledge which it should provide. Certainly this class needs the generous assistance given to it, and the new hospital should do something to provide the hospital treatment which the middle classes now find it so hard to procure. But it is interesting to note that the donor has devoted his foundation to a special purpose and not to the establishment of a general hospital for the class which he has in view. It would almost seem as if functional nervous disorders peculiarly preyed upon the middle class, and the public would welcome a more complete explanation why neurasthenia, in its broad sense, should be a middle-class disease. Grateful as one must be for this latest benefaction, it yet seems that the hospital problem for the middle classes requires for its solution general hospitals, since the establishment of an adequate number of paying wards at existing institutions must wait till better times arrive.

### HOSPITAL HORTICULTURE IN SUSSEX.

Unlike German hospitals, our own, except some institutions for consumptives, incurables, and mental patients, do not much study their gardens or rely much upon their own produce. It is interesting to hear, therefore, that the Royal West Sussex Hospital, Chichester, has purchased an adjoining meadow of three and a-half acres in order to supply itself with potatoes and other vegetables. We hope to hear how the scheme succeeds, and to study the cost and returns of this departure.

### RETIREMENT OF DR. BEDFORD PIERCE.

Dr. Bedford Pierce, who has decided to retire at the end of the year from the position of medical superintendent of the Retreat, York, has been for nearly thirty years associated with that institution. He has worthily maintained the traditions of a place which is honourably linked with the reform of the treatment of the insane. The Retreat was founded in 1796 by William Tuke: and it was to provide for "a milder and more appropriate system of treatment than that usually practised." Dr. Bedford Pierce was appointed to the staff in 1892, after a distinguished career as a student, and after being for a time clinical assistant at Bethlem Hospital and at the Edinburgh Royal Asylum. Dr. Pierce has been a considerable writer on his own branch of work, and has for some years past contributed the *précis* of psychiatry to the "Medical Annual." He has been president of the Medico-Psychological Association, and has taken a deep interest in the training and welfare of asylum nursing staffs. He is one of the representatives appointed by the Minister of Health on the General Nursing Council for England and Wales. Dr. Pierce is to be succeeded by Dr. Henry Yellowlees, who is at present deputy physician-superintendent of the Royal Edinburgh Asylum, Morningside.

### THE EFFECT OF CLOSING BEDS.

The Finance Committee of King Edward VII. Hospital, Cardiff, has had to recommend no extension of expenditure. At the same time—and other hospitals should note this point—Mr. W. J. Mansfield has urged that the remaining available beds should be put in commission, because the present long waiting-list tells against the hospital financially. People, he remarked, will not contribute to a hospital to which their friends cannot obtain admission. At present the workmen of Swansea contribute £23,000, or one-third more than the workmen of Cardiff. It is proposed to approach each firm in turn to remedy this, and especially the smaller firms, which seem to be the most backward.

